# Nurses’ experiences of accompanying patients dying during the COVID‐19 pandemic: A qualitative descriptive study

**DOI:** 10.1111/jan.15195

**Published:** 2022-03-21

**Authors:** Anna Castaldo, Maura Lusignani, Marzia Papini, Stefano Eleuteri, Maria Matarese

**Affiliations:** ^1^ IRCCS Foundation Don Gnocchi Milan Italy; ^2^ Department of Biomedical Sciences for Health University of Milan Milan Italy; ^3^ Faculty of Medicine and Psychology Sapienza University of Rome Rome Italy; ^4^ Campus Bio‐medico University of Rome Rome Italy

## Abstract

**Aims:**

The aim was to explore nurses’ experiences of caring for and accompanying patients dying without the presence of family during the COVID‐19 pandemic.

**Design:**

A qualitative descriptive design was used.

**Methods:**

A purposive sample of registered nurses, caring for dying patients in Italian health‐care settings during the COVID‐19 pandemic, were selected. To capture a broad range of experiences, a maximum variety of participants about workplace, age, gender and work experience were included. A total of 31 nurses agreed to participate in the study; 28 participants were female; participants were aged 41 on average (range 25–63 years) and worked in hospitals and nursing homes in the Northern and Central Italian regions. Six focus groups were conducted from August to December 2020 through Microsoft Teams, reaching data saturation. The transcripts were analysed through inductive content analysis. The COREQ checklist was followed for study reporting.

**Results:**

Four main categories emerged describing nurses’ experiences: hugely increased deaths in time of COVID‐19; ensuring physical, emotional, interpersonal and spiritual care for dying patients and supporting their families despite difficulties; ensuring care procedures of patients’ bodies after death and psychological consequences of caring for dying people during the pandemic.

**Conclusion:**

Registered nurses provided physical, spiritual and emotional care to dying patients during the pandemic, despite limited resources and knowledge, standing in for relatives who were not allowed to visit them; the experiences of caring for dying patients in the absence of the family caused emotional distress in nurses.

**Impact:**

Health‐care systems should develop guidelines aiming to meet the psychological needs of dying people in case of restrictions on patients’ family visits. Health‐care administrators should offer services to support the grieving process of relatives who cannot accompany dying patients and to reduce emotional distress in nurses caring for dying patients.

**Brief summary**

The experiences of accompanying dying patients were highly traumatic for interviewed nurses; however, despite all the difficulties characterizing the first wave of the COVID‐19 pandemic, they provided the best possible care for dying patients, replacing their relatives at the patients’ bedside and accompanying them as they died.

## 1 INTRODUCTION

Worldwide, nurses have had a pivotal role in caring for patients with COVID‐19. Due to the high number of casualties occurring in a short time (World Health Organization [WHO], 2021), and the need to prevent the spread of infection, nurses had to modify end‐of‐life care and the usual practices associated with patients’ deaths (Montgomery et al., 2021). Nurses felt alone and inadequately prepared to accompany so many people when dying, without the presence of their relatives, and with no time to process their own grief (Rabow et al., 2021). Caring for patients during the COVID‐19 pandemic had a significant impact on nurses’ mental health (Ruta et al., 2021). High levels of stress, depression, anxiety, sleep disturbances and post‐traumatic stress disorder (PTSD) were reported globally, related mainly to fear of contracting the disease and infecting family members, long and stressful working hours (Ruta et al., 2021) and repeated exposure to patient suffering (Kunz et al., 2021).

### 1.1 Background

The Severe Acute Respiratory Syndrome Coronavirus‐2 (SARS‐CoV‐2) infection caused a pandemic that has affected every country in the world (WHO, 2021), undermining their health‐care systems. It also caused numbers of deaths surpassing those in other pandemics occurring in recent decades, such as the Severe Acute Respiratory Syndrome (SARS) in 2002 and Middle East Respiratory Syndrome (MERS) in 2015, Swine Flu in 2009 and Ebola in 2014, which caused thousands of deaths (Pitlik, 2020). By the end of October 2021, the deaths caused by COVID‐19 worldwide were 4,979,421, including 1,418,911 in Europe and 131,004 in Italy (WHO, 2021), one of the first countries in Europe to be dramatically affected by the SARS‐CoV‐2 infection (Livingston & Bucher, 2020). Due to the highly contagious nature of COVID‐19, its lethal effects and the lack of of personal protective equipment (PPE) in many countries, in health‐care settings, preventive restriction measures have been applied, including restrictions or complete prohibition of families visiting the patients (Miralles et al., 2021). Consequently, dying patients in hospitals and in nursing homes were not able to see their families or friends (Kunz et al., 2021); thus, many patients died alone without the support of their loved ones. The COVID‐19 pandemic has, therefore, disrupted the usual practices of family members accompanying the dying person (Bermejo, 2020) and the mourning rituals, such as saying goodbye, viewing and burial of body (Eisma et al., 2020). Moreover, the COVID‐19 pandemic has modified the practices that health‐care personnel performed to care for dying patients, including ensuring the presence of the family at patients’ bedsides (De la Rica Escuín et al., 2020). As previously reported after outbreaks when death‐related rituals are disorganized and postponed, bereaved next‐of‐kin can suffer from psychological distress, such as PTSD and protracted or complicated grief manifested in anger, guilt and other symptoms of emotional pain that last over 6 months after the loss (Eisma et al., 2020).

A few qualitative studies have been conducted to explore nurses’ experiences of caring for patients during the COVID‐19 pandemic, reporting that inability to provide comfort, experiencing patient deaths, sense of isolation, concerns about PPE, care delays and changed clinical practice guidelines impacted their physical, emotional, psychological and social well‐being (Gordon et al., 2021; Montgomery et al., 2021). Despite the effects on their physical and emotional well‐being, nurses were willing to work and take care of patients (Villar et al., 2021) and develop resilience, meaning the ability to adapt to changes caused by stressful events and to recover from negative emotional experiences, using social support and self‐regulation strategies (Huang et al., 2021). To our knowledge, no qualitative studies have been conducted to investigate nurses’ experiences of caring for dying people during the COVID‐19 pandemic and of accompanying dying patients without the presence of the family. Knowledge of these aspects can provide an understanding of what strategies are implemented by nurses during pandemic events to guarantee patients’ end‐of‐life care and to support relatives in coping with their loss; moreover, it can provide more insights on how nurses cope with their own grief at the loss of their patients.

## 2 THE STUDY

### 2.1 Aim

This study aimed to explore nurses’ experiences of caring for people who were dying and of accompanying those dying in health‐care settings without the presence of their family, during the COVID‐19 pandemic.

### 2.2 Design

We used a qualitative descriptive design since it enables researchers to produce a direct description and complete summary of the phenomenon under study using the language of participants and remaining close to the data (Sandelowski, 2000). To assure the quality of reporting the consolidated criteria for reporting qualitative studies (COREQ) checklist was used.

### 2.3 Sample/participants

Registered nurses who cared for dying patients in Italian hospitals or residential care facilities during the COVID‐19 outbreak were included. We used a purposive sample of nurses who cared for dying patients during the pandemic in settings where the presence of patients’ families was restricted, to provide information relevant to the research topic (Polit & Beck, 2017). To capture a broad range of experiences, we aimed to include a maximum variety of participants with regard to Italian regions (North, Central and South Italy), workplace (COVID hospital units, non‐COVID hospital units and nursing homes), age (21–34, 35–50 and >51), gender (female and male) and years of work experience (0–5, 6–10 and >10), as these factors could influence how nurses experience patients’ death (Cardoso et al., 2021). Registered nurses were recruited through professional and scientific associations by e‐mails, advertisements on their websites and telephoned requests to nurses belonging to these associations to suggest other colleagues who cared for dying patients during the pandemic and met the characteristics listed, to achieve maximum variation. A total of 31 registered nurses agreed to participate in the study and met the inclusion criteria. Most of the participants were female, with a mean age of 41 years and 15 years of work experience (Table 1).

**TABLE 1:** Characteristics of focus group participants

Variables	*N* (%)
Age (years) Mean ± SD (range)	41 ± 11.74 (24–63)
24–34	12
35–50	12
>51	7
Working experience (years) Mean ± SD (range)	15.37 ± 11.92 (1–38)
0–5	9
6–10	7
>11	15
Gender	
Male	3 (9.7)
Female	28 (90.3)
Nationality	
Italian	30 (96.8)
European union	1 (3.2)
Italian regions	
North Italy	
Lombardy	25 (81)
Piedmont	1 (3)
Trentino‐Alto Adige	1 (3)
Emilia‐Romagna	1 (3)
Central Italy	
Abruzzo	1 (3)
Latium	2 (7)
Qualifications	
Nursing degre	25 (81)
Professional diploma	6 (19)
Post bachelor education	
Master of Nursing Science	4 (33)
Clinical Master’s degree	8 (67)
Health‐care setting	
Hospital	28 (90.2)
COVID unit	20 (71)
Non‐COVID unit	8 (29)
Nursing home	3 (0.98)

Abbreviation: SD, standard deviation.

### 2.4 Data collection

Focus group interviews were used as they enable rich, deep data to be generated through participants’ interactions, facilitating self‐disclosure on sensitive topics (Freeman, 2006). An interview guide was developed based on researchers’ experiences, including opening questions, questions encouraging discussion and summary questions (Polit & Beck, 2017) (Table 2) and pilot tested on the first focus group; no subsequent amendments were needed to the interview guide, as the participants’ responses met the scope of the study, and therefore, the pilot focus group was included in the analysis. The focus groups were conducted online through Microsoft Teams (Redmond, WA) to ensure social distancing measures. From August to December 2020, six focus groups were conducted, scheduled at different times to adapt to nurses’ shifts and facilitate the nurses’ participation. All 31 nurses participated in the focus groups, with a mean of five participants per group (range 2–10), as literature suggests (Freeman, 2006); no nurses refused to participate or dropped out. The participants could choose whether keep the webcam on or off and put a neutral background on the screen if they did not want to show where they were. It was also suggested to find a reserved space at home or in the workplace to participate in the discussion without interruptions. We asked participants not to mention the name of the health‐care setting they worked in to ensure privacy. The average duration of focus groups was 95 minutes, with a range of 75–116 minutes. During the focus groups, the participants could have asked for a break if they needed it, but they chose not to interrupt the discussion. Focus groups were audio‐video recorded after obtaining participants’ consent. Two nurse researchers conducted the focus groups; one, experienced in focus group conducting, acted as a moderator (AC), and the other, an expert in end‐of‐life care, as an observer (MP) to record non‐verbal communication and interactions among participants through field notes. In case of participants becoming distressed during the interview, the researchers planned to allow them to express their feelings, create an atmosphere of compassion and empathy and suggest leaving the session if they wished. Interaction among participants was also facilitated by the fact that all participants kept the webcam on for all the duration of the focus groups.

**TABLE 2:** Focus group interview questions

During the coronavirus pandemic, have you ever taken care of and accompanied dying people, in the absence of a family member?
Can you tell us your experience? How did you feel when you cared for dying people who could not say goodbye to their loved ones? Would you like to tell a story about your experience?
What kind of strategies did you use to deal with the absence of relatives? In your opinion, which strategies were more useful? In your opinion, what were the factors that facilitated end‐of‐life care and farewells in the first wave of the pandemic? In your opinion, what were the factors that impeded end‐of‐life care and farewells in the first wave of the pandemic?

Four members of the research team (AC, ML, MM and MP) were female nurses working in nursing schools and experienced in conducting qualitative studies, and the fifth member (SE) was a male psychologist working in a nursing home. Two researchers (AC and SE) had PhDs (Life Cycle Welfare Sciences and Psychology and Educational Studies, respectively), and the rest had Master’s degrees in Nursing Science. Participants’ sociodemographic and professional data were collected before focus group sessions through a Google Form questionnaire to guarantee the data confidentiality; the data were available only to two researchers (AC and MP). Data saturation, shown by no new information emerging from the data, was verified at the end of the planned six focus groups including all the nurses who agreed to participate in the study. As data saturation was reached after the third focus group, no further recruitment of participants was needed (Kerr et al., 2010). The saturation grid is reported in Table S1.

### 2.5 Ethical considerations

The study was conducted in accordance with the Declaration of Helsinki. The Ethics Committee of a health‐care institution approved the study (number 2020_07_01). Before each focus group was held, each participant was informed by e‐mail or telephone about the study aim and procedures; if they agreed to participate, a written consent form was sent by e‐mail to be signed. All participants were instructed to keep all the information shared by other participants confidential and to avoid reporting during the interview, the names or details of patients and other people involved in their stories which could lead to their identification. Anonymity was guaranteed by using codes to identify participants on the transcripts, in data analysis and in the study report.

### 2.6 Data analysis

The focus groups were transcribed verbatim. Inductive content analysis was used to analyse the data in four steps. First, transcripts were read several times to obtain a sense of the whole. Second, codes were generated by reading the transcripts word by word. Then, codes were organized into subcategories based on their similarities and differences. Finally, the subcategories were grouped into categories based on their commonality (Graneheim & Lundman, 2004). The field notes were not analysed as the video recordings allowed observing the non‐verbal communication and interactions between the participants during the transcriptions. Transcripts were exported into Atlas.ti version 7.5.7 to be managed, organized and coded. Two researchers (AC and MP) coded and categorized the data separately. Before starting the analysis, each researcher declared their beliefs about the study topic, to reduce possible bias in the data interpretation. The codes, subcategories and categories proposed by the researchers were compared, and any divergence was resolved through discussion. A third researcher (MM) checked the codes and categories for consistency, and any discrepancy was discussed by the research team and resolved through discussion and spot‐checking of the transcriptions, until consensus was reached.

### 2.7 Rigour

To ensure trustworthiness, criteria for credibility, confirmability, dependability and transferability were considered (Graneheim & Lundman, 2004). Study credibility was assured by prolonged data engagement, independent data analysis and peer debriefing. For confirmability and dependability, an audit trail was kept during the whole study process, supervised by a member of the team not involved in data analysis. Member checking was carried out by video call involving 18 participants who confirmed that the findings reflected their experiences. Transferability was ensured by providing details about the study participants, the context where the research was carried out and a thick and rich description of the research process and findings.

## 3 FINDINGS

A total of 88 codes were derived from the transcripts, grouped in 18 subcategories; from these, we derived four categories that describe the nurses’ experiences of caring for and accompanying patients dying during the pandemic in the absence of their family: (1) unprecedented deaths in the time of COVID‐19; (2) ensuring physical, emotional, interpersonal and spiritual care for dying patients and supporting their family despite the difficulties; (3) ensuring care procedures for patients’ bodies after death; (4) psychological consequences of caring for dying people during COVID‐19 pandemic (Table 3).

**TABLE 3:** Description of categories, subcategories and codes

Category	Subcategory	*Code*
Unprecedented deaths in the time of COVID‐19	Large‐scale death	Freewheeling death Fast patient turnover due to death High number of deaths Death Shared in open space
	Unpredictable death	Death from sudden deterioration Death of the young and older people
	Death in isolation	Death in protective isolation Death in solitude
Ensuring physical, emotional, interpersonal and spiritual care for dying patients and supporting their family despite the difficulties	Providing end‐of‐life nursing care to patients	Providing bodily care to dying patients Assisting the dying patient with competence and compassion Preparing the patient for death Staying after hours of service next to the dying patient Encouraging the patient to eat Caring for the patient's body for a dignified death
	Providing emotional support to patients and their families	Caressing the patient Comforting Drawing smiles on the masks Expressing closeness Managing the patient’s grief for the death of a family member Positive presence of the relative for patient and staff Relationship with the patient limited by PPE Embracing the patient Emotionally supporting long‐distance family members Reassuring the patient Asking for psychological support for family members for bereavement process
	Facilitating last communications between patients and their family	Identifying the best time for communication Showing the dying patient from the unit window Interrupting CPAP to allow communication with the relative Allowing the entry of relatives with PPE in the COVID unit Allowing the entry of relatives with PPE in non‐COVID units Last goodbyes between patient and family member via telephone (audio and video calls) Evaluating when last goodbyes via video call is appropriate Participating in the last goodbyes by video call
	Being the liaison between patient and family	Being the patient’s voice Acting as a messenger between patient and family Acting as an intermediary between patient and family Acting as a bridge between patient and family
	Replacing the family in the last farewell	Exchanging the last words with patients Patient’s death perceived as the death of one’s family member Considering the patient as a family member Replacing relatives at the end of life Being close to the patient as a friend, a relative
	Ensuring spiritual support to patients	Praying for the patient Praying with the patient Absence of spiritual accompaniment Delegation from the priest for extreme unction Requesting extreme unction Asking for the spiritual support of a priest
Ensuring care procedures for the patients’ bodies after death	Participating in informing of the patient’s death	Giving news of patient’s death by phone Showing relatives the deceased via tablet
	Performing rapid procedures for ascertaining death and preparing the body	Modification of the body preparation practices Modification of the practices of death ascertaining
	Managing the patient’s belongings after death	Personal effects of the deceased thrown away by family members Destruction of personal effects Loss of personal items in transfers between facilities Return of personal effects to relatives
Psychological consequences of caring for dying people during COVID‐19 pandemic	Suffering over the disruption of rituals associated with death	Modification of accompaniment modalities Modification of the rituals that accompany death Modification of palliation practices Suffering over the inability of relatives to see the patient Suffering over the ways of preparing the body
	Suffering from moral distress	Deciding to remove oxygen from one patient to give it to another Deciding which patients to resuscitate Deciding which patients to admit to ICU Deciding on the distribution of resources Inability of family members to see the deceased Transferring dying patients to clear ICU beds Reducing care practices due to lack of time Sense of despair for lack of resources
	Suffering from emotional distress	Difficulty in relating to dying patients Strong emotional involvement Knowing about the family members’ death after days Poor information about the patient / deprived of identity Sense of frustration Sense of inadequacy Sense of helplessness Suffering in remembering Suffering in attending the farewell between patient and family Inhumane death
	Experiencing unprocessed grief	Unprocessed emotions Failing to process mourning Unexpressed pain ‘You cannot bear so many deaths at once’
	Empathizing with patient and family	Identification with patients Identification with patient families
	Finding a positive meaning	Giving a transcendent meaning to the procedures of body care Feeling a clear conscience for having enabled goodbyes between family members and patients Counting on the mutual support of the health‐care team

Abbreviations: CPAP, continuous positive airway pressure; ICU, intensive care unit; PPE, protective personal equipment.

### 3.1 Unprecedented deaths in the time of COVID‐19

According to participants, death caused by COVID‐19 presented some characteristics that profoundly influenced the practices of accompanying dying patients, including the high death toll, unpredictability and deaths in isolation.

#### 3.1.1 Large‐scale death

Nurses witnessed the death of very many people in a short time, a *freewheeling death that mowed down everyone (focus group [FG]1, Female [F], COVID Intensive care Unit [ICU]*). It was an unprecedented experience, even for nurses working in hospices or intensive care units, who were more used to dealing with patients’ deaths:

You can try to put up with seeing a person die every now and then, in the sense that it is part of life, but seeing so many people die every day was a quite devastating thing, especially when they were alone (FG2, F, COVID Medicine).

#### 3.1.2 Unpredictable death

Death from COVID‐19 was neither a sudden death, as happens from a heart attack, nor a slow death as in oncological diseases; several days or weeks could pass between the time of COVID infection and the patient’s death. Health could worsen suddenly in younger as well as in older patients who seemed to be recovering, so death was often unpredictable:

Many people seemed to be doing well, then in fact they did not make it because this disease was also quite insidious; it had moments of ups and then moments of unforeseen downs, even in people for whom you would not have expected it (FG1, F, COVID ICU).

#### 3.1.3 Death in isolation

Patients with COVID and patients with other pathologies were all in isolation, resulting in restricted access for family members in all health‐care settings; this led to patients dying alone, without the comforting presence of family members.

### 3.2 Ensuring physical, emotional, interpersonal and spiritual care for dying patients and supporting their family despite the difficulties

When a patient’s death was predictable, nurses provided the needed care, including physical, emotional, interpersonal and spiritual care; facilitated last communications with family and replaced family at the moment of death.

#### 3.2.1 Providing end‐of‐life nursing care to patients

Many nursing activities were directed to the bodily care of dying patients; it was important for nurses that patients were clean and tidy. Through bodily care, nurses, along with other health‐care professionals, demonstrated their respect for the dying person and tried to guarantee care preserving the patient dignity in the last phase of their life:

There is no patient who died badly, abandoned or dirty, that is, no one died dirty. I guarantee you, all patients we cared for were perfumed, clean and perfect (FG1, F, COVID ICU).

The nurses provided competent and compassionate care to the dying patients. They also remained beyond their working hours to be sure that all patients received the needed care:

Many remained next to the older people in the terminal phase, even staying beyond their hours of service because they knew the older patient's relatives or because they were fond of the patient (FG6, F, nursing home).

#### 3.2.2 Providing emotional support to patients and their family

Nurses spent a lot of time with patients and tried not to let them feel alone despite family absence. They understood patients’ suffering at not having their loved ones next to them, even for a quick visit; they replaced their family, expressing joy for their small improvements, distracting them by joking, showing closeness and affection by holding their hands, caressing and hugging them:

There was this man with these blue eyes who 1 day told me ‘Hug me’, and the hug was the only thing I could give him (FG2, F, COVID medicine).

Despite the presence of gloves, overalls, visors and masks that made communication difficult, nurses tried not to let patients feel they were among strangers; they drew smiles on their masks and wrote their names on their overalls. There were cases in which entire families got the infection, and nurses comforted the patients when they received the news of their loved ones’ death.

There was this boy, my age, whose mother was hospitalized in another unit, and who received a call announcing the death of his father in another hospital, and at that moment, he needed support (FG2, F, COVID unit).

On rare occasions, a visit from a family member, wearing PPE, was permitted, providing emotional support to patients, but also relieving nurses from the emotional burden of being the only ones to accompany patients as they died.

Nurses also provided emotional support over the telephone to patients’ families despite the difficulties due to distance.

We also tried to help families experience this moment in the least painful way possible. Although I must say that virtual grief has put a strain on all health‐care providers: none of us were prepared to handle such a crippling situation (FG2, F, hospice).

#### 3.2.3 Facilitating last communications between patients and their family

Given the visiting restriction, nurses found alternative ways to enable communication between patients and their families when the patients got worse, before sedating or intubating them, and even when the patient was stable as the disease evolution was unpredictable.

I remember a very old man, […] and I remember that before starting palliative sedation, we helped him say goodbye to his family. I spent 2 or 3 days with him, I set up a video call with his sister who was the only relative left. Here, in short, first, the physician spoke to the family, explained the situation to him, and then I helped him make the last video call, and then I started the palliative sedation (FG5, Male [M], COVID ICU).

Several strategies were applied to facilitate patients’ communication with families: for example, stopping respiratory supports and checking if they could handle a conversation.

When conditions allowed, family members could see the dying patient from the windows or balcony of the unit. However, the most common strategy reported to allow the last farewell between patients and family members was by phone or tablet, through audio and video calls, ways unthinkable in normal times. At first, the nurses used their cell phones to enable patients to communicate with family members; later, the units were provided with tablets, which were used above all for the most serious patients or the older ones without personal phones. During conversations with families, nurses helped patients to manage the devices and spoke for them when they were unable to do it; thus, nurses found themselves, unwittingly, listening the conversations, but trying to respect their privacy.

Nurses allowed family members to say goodbye to their loved ones through video calls even when the patient was not fully conscious, to respond to the family's need:

A son from Switzerland asked us to put his mother on the phone even though we didn't know whether at that moment she could hear her son because she was in a serious condition. He stayed on the phone with his mom for 15–20 minutes talking to her all the time and shortly afterwards she died. And so, we tried to show our closeness to the relatives too in this phase (FG6, F, nursing home).

Video calls were not always easy because some family members could not endure the sight of their loved one, especially when their appearance changed due to treatments; for this reason, nurses preferred audio messages and phone calls:

Regarding the family, I understand that someone would like to see them via tablet. But the management of death is not always simple, so seeing your dad and mom suffer through a tablet… was not always the best. And I understand this great suffering (FG3, F, COVID unit).

#### 3.2.4 Being the liaison between patient and family

Due to the impossibility of families visiting dying patients, even in non‐COVID units, the nurses acted as messengers between relatives and patients. They handed patients messages, clothes, food, photos and personal items brought by family members:

It happened, especially for older people, that grandchildren and children brought letters, photos, and wanted us to put them near their bed, and this was very touching, even if the person was not conscious, but for them, it was important […]. Or they sent us by phone the photo of the grandchildren, of the family, and they wanted us to show them to the patient; and many times, when we showed the photos of their loved ones, even if patients were not fully conscious, they reacted, a tear fell, and there you were moved too (FG5, F, neurology unit).

#### 3.2.5 Replacing the family in the last farewell

The nurses gave comfort and expressed closeness to patients, taking the place of family members for the last goodbye:

We always took the time to say goodbye to them replacing the family; and although it was distressing and terrifying that it was a stranger to say goodbye to them, because family couldn't see them even after death, it was certainly a positive thing, having the opportunity to say goodbye to them in their place even if it was not the same thing (FG6, F, COVID ICU).

Some nurses claimed that the patients did not die alone since they were there holding their hands and consoling them. For the patients, nurses were their new family members, but for nurses too, the patients became family members.

It bothered me a bit when I heard people say on TV ‘these people died alone’. It's true they didn't have their family next to them, but we were there. I mean, almost all of us treated them as if they were our father, our brother, our uncle (FG1, F, COVID ICU).

#### 3.2.6 Ensuring spiritual support for patients

When possible, nurses made sure that patients received spiritual support by requesting the presence of a priest, especially to receive extreme unction.

I realized that person was dying and I said ‘did we call the Father?’, that is, because I am from the oratory and therefore it's normal for me to say "has the Father come?". I say the Father, but I mean ‘did this person have some accompaniment at the spiritual level?’ (FG1, F, COVID ICU).

Sometimes, the priests were not allowed to enter the units; other times, the priest himself got sick and was no longer able to visit patients and the health‐care personnel was delegated to administer the last rites. Nurses prayed for and with patients when they believed it was necessary for the patients but also for themselves.

### 3.3 Ensuring care procedures for patients’ bodies after death

Nurses participated in breaking news of death and followed rapid post‐mortem practices because beds had to be freed quickly for other patients, and bodies had to be decontaminated before moving to the morgue.

#### 3.3.1 Participating in informing about patients’ deaths

Although the news of a patient’s death was given by telephone by physicians, nurses witnessed the grief of families and other health‐care professionals:

And I saw physicians cry. Above all, it was tragic to hear the sobbing and crying of family members on the phone when they were notified of the patient's death, or that there was nothing more to be done, both for the older and non‐older patients (FG2, F, COVID ICU).

Some family members requested to see their loved one’s body via a tablet to begin their grieving process.

The wife of a 52‐year‐old man asked us to see his body. And then, we made a video call between the wife and her husband who had passed away […]. It was a courageous act but necessary, to process what was happening, but very hard on our part, I mean, it was really difficult for us to take that tablet and keep it pointed towards the lifeless body of this man while witnessing the suffering of his wife (FG2, F, COVID medicine).

#### 3.3.2 Performing rapid procedures for ascertaining death and preparing the body

The need to free beds for other patients waiting for admission changed existing procedures for ascertaining death and moving the deceased body from the unit:

At the beginning, according to regulations, they stayed in the unit 2 hours under observation in order to confirm death; afterwards, the indication came to free the beds as quickly as possible, within a maximum of 20 minutes from the moment of death, after 20 minutes ECG (FG5, M, COVID ICU).

The usual procedure for preparing the body after death changed, especially in the 1st month of the pandemic, as the survival capacity of the coronavirus was unknown. The directives were to wrap the body in sheets, sprinkle it with sodium hypochlorite and place the body in airtight bags or in a zipped double plastic bag. As it was impossible for the mortuary staff to enter the unit, the nurses also had to transfer the patient from the bed to the metal mortuary stretcher and leave it outside the unit for collection by the mortuary staff. Even for patients dying in non‐COVID units, the same procedure was followed as the patient had to be considered potentially infected in the absence of a negative COVID swab. This made it impossible for family members to see the loved one after death or stay with the body in the mortuary room.

#### 3.3.3 Managing patients’ belongings after death

After patients’ deaths, nurses put personal effects in plastic bags and stored them in a designated room, awaiting collection by families. Sometimes, they were stored for weeks as no one claimed them.

There was this room where we stored the effects of the deceased […], there were piles and piles of bags, one on top of the other, all bags of people who died. Later, they equipped it with a large safe, and it was practically full of ID cards, wedding rings, mobile phones. And every now and then, the bad thing was that you heard these phones ringing inside the safe and you knew it belonged to a dead person and that someone was looking for them (FG5, M, COVID ICU).

The moment of handing over belongings to family was very difficult for both family and nurses, who were unable to find the words to console them.

The way to deliver their personal effects was also bad; to avoid contagion, we closed them in sacks and we handed them to relatives at the unit door. And as soon as they received these sacks, they got down on their knees and cried. That was it, we felt helpless, but in the end, the only thing to do was remain silent, because there was nothing to say (FG2, F, COVID medicine).

Sometimes, the belongings, according to local directives, were burnt because considered infected; other times, relatives refused to take them home for fear they were infected.

The thing that really struck me was seeing the patients’ possessions thrown into the bin because the relatives didn’t want to take them home out of fear. And you see the objects of a person’s life that go away with the person. I mean, it’s something touching you, especially for people who had lived in the nursing home for years, and that was their home. And you saw that a lot of their photos… all their things were thrown away, and nothing remained to the family (FG3, F, nursing home).

### 3.4 Psychological consequences of caring for dying people during COVID‐19 pandemic

The experiences of caring for dying patients and the difficulties in providing adequate accompaniment whilst dying had a deep impact on nurses. Profound suffering over the disruption or disorganization of rituals associated with death, moral distress caused by difficult choices and lack of time to mourn for the loss of patients left an indelible mark, even though some of them managed to give a transcendental meaning to their experience.

#### 3.4.1 Suffering over disruption of rituals associated with death

Disruption of personal and professional rituals associated with patients’ deaths caused deep suffering in nurses. None of the usual bodily care after the death was permitted: washing the body, wrapping them in clean sheets, putting nice clothes on them and placing the body in a room for the family wake. When patients would have needed their loved ones most, when they were alone and scared, the family was not allowed to be with them or to see them for the last time, even after death.

The thought that a mother doesn't have her child next to her in such moment… it's like birth, birth and death are two fundamental things, the mother gives birth to her child and then the child accompanies her as she dies, it is a natural thing. The thought that a family member can't do anything is dreadful, I mean, you felt bad (FG4, F, COVID unit).

#### 3.4.2 Suffering from moral distress

Nurses described the suffering caused by not being able to act according to their professional and personal values, especially in the 1st month of the pandemic. They had to decide, together with the medical staff, about the allocation of resources in time of shortage, the suspension of family visits for dying patients, which patient to provide care to and which one to remove it from, causing intense moral distress.

We dealt with a dramatic choice, that of removing oxygen from a person in terminal phase, in his last hours, to give it to a person who had recently manifested symptoms and could have some chance of surviving. The physician said openly, crying ‐ and if I think about it I still feel like crying ‐ that she had chosen to become a physician not to take a resource from one person to another; in any case, she shared the choice with us, but in the end, it was me who went to remove the oxygen from that person to give it to the other, so this thing was very difficult (FG6, F, nursing home).

#### 3.4.3 Suffering emotional distress

Nurses felt inadequate to respond to the needs of many dying patients, helpless and frustrated at the thought of not being able to do enough.

It's a huge emotional burden because, out of the 30 patients you care for, you know all of them, you take home all 30, because it's impossible not to take your work home. They say to separate one's personal life from the professional one, but nurses are really screwed because it's impossible to do this in this historical moment (FG6, F, COVID ICU).

Personally having to manage the last farewells between patients and relatives via video call caused additional suffering to nurses.

Emotional distress was also generated by not having time to know patients as persons, especially if they were unconscious. The person’s identity was all in the plastic bag containing their personal items.

The other bad thing is this, that it was all so fast that you didn't know anything about the patient, you couldn't know anything, or when you could, you read it from the medical records, but it was all medical information anyway; I mean, it didn't say what their religion was, how many children they had, what they did in life (FG1, F, COVID ICU).

Dying without the presence of the family and the relatives’ wake was considered inhumane.

Maybe I use a wrong and even a strong term, but [death] was inhuman, that is, inhumane because you didn't even have time to accompany them (FG1, F, COVID ICU).

Another source of suffering for the nurses was not being able to share with family members the patients’ last moments, to have the chance to tell them what they had said and done. Contacts with relatives, when there were any, were mainly by phone, and after a patient’s death, they had no opportunity to meet them.

#### 3.4.4 Experiencing unprocessed grief

Double shifts, high patient turnover and numerous deaths did not allow nurses to express grief for patients’ deaths or mourn for them, although some health‐care facilities offered psychological support to staff.

It was a missed grief for everyone […] While a patient was dying in a room, there was the anaesthetist ready to intubate someone else. I mean, we were not given time to process what we were experiencing, […] it was right to do so, but the repercussions of this lack of elaboration in the long term will be many. I can't talk about it with my friends who aren't nurses, and with my friends who are nurses we don't talk about it anymore. It's as if we wanted to remove that thought from memory as much as possible (FG1, F, COVID ICU).

#### 3.4.5 Empathizing with patient and family

Nurses often identified with patients and their families and acted thinking about what they would like for themselves or their own loved ones in that situation, finding a reason to move forward despite the suffering.

This woman, who passed away when I was there, pulled something out of me that wasn't mine and I'll tell you why: this woman looked a lot like my mother [trembling voice, and shining eyes]. And my mother is old, and the thought that it could happen to her, she gave me a strength that is not mine; many of us have parents in the South [Italy], and the thought that there could be a person who looked after my mother as if she were her mother gave me that strength (FG4, F, COVID unit).

#### 3.4.6 Finding a positive meaning

Nurses tried to find a transcendent meaning to what they were experiencing to alleviate their suffering, especially in cases of disturbing practice.

I had to find my own meaning to pour this disinfectant on the bodies. I imagined that liquid was light, light for their soul because it was a struggle to pour it… if they'd been my relatives, I couldn't have poured this on them, right? […]. So, I visualized that I was giving light with this liquid, so the black plastic bag became light for the soul of the person. And when I gave dignity to all this in this way, for me it was not a burden, but a joy and a responsibility (FG3, F, COVID unit).

The emotional distress experienced by nurses was alleviated by the creation of a strong sense of solidarity among the health‐care team. The common purpose and mutual support helped to cope with the situation.

A very positive thing was to work in a team, the teamwork, because we really found ourselves in critical situations with a lack of staff and we really joined in beyond the roles. Everyone did what they could (FG3, F, nursing home).

## 4 DISCUSSION

The aim of this study was to explore the experiences of nurses who cared for and accompanied patients dying without the presence of family during the COVID‐19 pandemic. Our findings showed that the COVID‐19 pandemic modified the physical, interpersonal, emotional and spiritual care provided by nurses to dying people and the care of the patients’ bodies after death; these modifications had an impact on nurses’ psychological well‐being. Several strategies were used by nurses to minimize and manage their grief and to help patients’ relatives to cope with their loss.

Our findings could be useful to nurses for the care of dying patients when relatives’ access to health‐care facilities is limited, in case of future pandemics as well as in normal times. They show that alternative ways of communication can be used to ensure daily contact between patients and family members. Tablets, smartphones and video calls give the oppourtunity to say goodbye to the loved ones and also nursing interventions can be performed to preserve the dignity of patients approaching death and after death, if the usual rituals cannot be performed. In addition, our study provides useful suggestions to health‐care administrators about the services that could be offered, even beyond the pandemic, to meet the physical and psychological needs of dying people’s family members and health‐care professionals, such as services that support relatives in their grieving process when they cannot stand by their dying family members and psychological services for health‐care professionals to reduce their emotional distress when caring for dying patients; changes could also be implemented in health‐care settings that allow relatives to see and talk to their loved ones safely, for example, by installing glass partitions or closed‐circuit television.

In Europe, during the first epidemic wave, Italy was the earliest country to be affected by the outbreak and had thousands of deaths (Livingston & Bucher, 2020). The restrictive measures applied in that country to reduce the risk of contagion in patients, health‐care providers and visitors led to the isolation of patients during their end of life, causing suffering in family and nurses. Although accompaniment whilst dying varies according to the culture and individual preferences, everyone is afraid to die alone (Nelson‐Becker & Victor, 2020; Wakam et al., 2020). Due to the large number of deaths in a short time and the need to reduce the risk of contagion, opportunities for social support and rituals related to death were limited. In health‐care facilities, where family visits were suspended, technology offered alternative forms of communication. Letters, phone calls and video calls were often the only chance for relatives to say goodbye to their loved ones. Even though these means could not replace face‐to‐face conversation and physical affection, they enabled families to make patients feel their affection (Selman et al., 2020), even when these options were challenging, such as with older people with cognitive impairment (Moore et al., 2020).

Our study showed that spiritual and religious needs were considered relevant by nurses. Even when patients were unable to express their religious needs, nurses became spokespersons for them or provided the last rites when needed. Nurses felt that their spiritual skills were particularly important during the COVID‐19 pandemic to relieve the stress and psychological suffering of patients and their families as well as of the nurses themselves (Chirico & Nucera, 2020).

Due to limited health‐care resources and restriction measures, the nurses interviewed struggled to provide care and preserve the dignity of dying patients in the absence of models of end‐of‐life care to apply during pandemics with patients in isolation. Chochinov (2002) has proposed a model of palliative care, named the dignity‐conserving care, which incorporates a broad range of physical, psychological, social and existential issues that may affect individual perceptions of dignity. Chochinov (2007) has suggested in his ABCD’s dignity‐conserving care framework what attitudes, behaviours and quality, including compassion and openness to dialogue, are needed to give dying individuals a sense of dignity during their approaching death. Research should be carried out to explore which interventions are most appropriate to preserve dying patients’ dignity during pandemics or other calamities when the usual practices cannot be performed (Chochinov et al., 2020). Being unable to ensure care whilst preserving the dignity of dying people, in coming years, could affect the way people, including nurses and relatives, process their grief after the critical phase of the pandemic. Some recommendations have been formulated to mitigate the negative effects on family members, including proactive, sensitive and regular communication with bereaved, providing accurate information and enabling them to say goodbye to their loved one when possible in person or through virtual communication. Moreover, the bereaved could benefit from emotional and spiritual support, information about services for the grieving process and support to adapt funeral rituals and bereavement to the situation (Selman et al., 2020). The UK government, for example, recognizing the importance of funerals for the well‐being of the bereaved, developed some regulations, including guidance for arranging or attending a funeral during the COVID‐19 pandemic (Chochinov et al., 2020). Community recognition and support for grief will require new approaches in the care setting (Moore et al., 2020). The large number of deaths, unpreparedness, stressful job conditions, helplessness because of scarce resources, lack of communication with families and lack of time to mourn, could lead to complicated grief. Nurses who experienced complicated grief in the future could present impairment in work, health and social functioning (Zisook & Shear, 2009).

Due to the pandemic, especially during the first wave, in many countries, practices for the care of the patient’s body after death were modified to avoid contagion. Performing the recommended decontamination of the body with disinfectants, such as sodium hypochlorite (Fineschi et al., 2020), was a devastating experience for nurses, and this experience will remain imprinted in their memory forever. For nurses, caring for people during the pandemic was a war‐like experience where, due to limited resources, they faced shortages of ICU beds and medical devices. This caused moral distress and sadness in situations where they were unable to act according to their beliefs, values and professional standards. They experienced moral conflict and ongoing grief when they saw their dying patients alone (Anderson‐Shaw & Zar, 2020). Moral distress and moral conflicts can seriously affect health‐care providers’ well‐being. National health‐care institutions and scientific associations have proposed some recommendations to ensure fair and ethical allocation of resources to help address the ethical dilemmas of this time (Anderson‐Shaw & Zar, 2020). Support from leadership and health‐care organizations is recommended to alleviate complicated grief and prevent negative long‐term effects on nurses (Selman et al., 2020).

### 4.1 Limitations

A few limitations must be acknowledged. First, the nurses’ experiences reported in our study referred to a specific country and a specific period of the pandemic, and therefore, the results can be transferred only to other countries where the COVID‐19 pandemic presented similar characteristics. The study took place at the end of the first wave of the COVID‐19 pandemic that occurred in Italy between February and May 2020 and at the beginning of the second one when the same restrictions on relatives’ access to health‐care institutions were applied. Second, the registered nurses participating in focus groups were mainly from the North of Italy and no nurse from Southern regions of Italy was recruited, leaving experiences from other Italian regions unrepresented; however, as in the first pandemic wave, these were the regions with the highest number of deaths, we believed this did not hamper the richness of data. Lastly, the focus group moderator knew some participants as they belonged to the same professional associations. However, given the nature of the topic, we believe that this facilitated the self‐disclosure of participants’ experiences.

## 5 CONCLUSIONS

This study provides an understanding of nurses’ experiences of caring for dying people during the COVID‐19 pandemic. Nurses used numerous strategies to meet the physical, relational, emotional and spiritual needs of dying patients, to manage their grief for the loss of the patients and to help patients’ relatives to cope with their loss. In the absence of families, nurses stood by patients during their last moments, even though this entailed a considerable emotional cost, with the consequences are likely to be visible in future years. Health‐care administrators should provide services that support the emotional and psychological needs of dying people, their loved ones and nurses and plan structural changes in the health‐care settings to allow the maintenance of relationships between dying patients and family members.

## CONFLICT OF INTEREST

No conflict of interest has been declared by the authors.

## AUTHOR CONTRIBUTIONS

All authors meet the criteria for authorship stated in the Uniform Requirements for Manuscripts Submitted to Biomedical Journals. Following there are listed all authors' specific areas of contribution:

- Study concept and design: Anna Castaldo, Maura Lusignani, Stefano Eleuteri
- Acquisition of data: Anna Castaldo, Marzia Papini
- Analysis and interpretation of data: Anna Castaldo, Marzia Papini, Maria Matarese
- Drafting of the manuscript: Anna Castaldo, Maria Matarese
- Critical revision of the manuscript for important intellectual content: Maria Matarese, Maura Lusignani, Stefano Eleuteri.

### PEER REVIEW

The peer review history for this article is available at https://publons.com/publon/10.1111/jan.15195.

## Supporting information


Table S1

Data S1
