# Supplementary material for: Nurses’ experiences of accompanying patients dying during the COVID‐19 pandemic: A qualitative descriptive study
Source: J Adv Nurs. 2022 Mar 21;78(8):2507–21. doi: 10.1111/jan.15195 (PMC9111619; doi:10.1111/jan.15195)
Supplement: Supplementary file 1 — Table S1 [file JAN-78-2507-s001.docx]

**Table S1** Saturation grid of subcategories represented in focus groups (alphabetical order)

| **SUBCATEGORIES** | **FG 1** | **FG 2** | **FG 3** | **FG 4** | **FG 5** | **FG 6** |
| --- | --- | --- | --- | --- | --- | --- |
| 1. Being the liaison between patient and family |  | x |  | x | x | x |
| 1. Death in isolation | x | x |  | x |  | x |
| 1. Empathizing with patient and family |  | x |  | x | x |  |
| 1. Ensuring spiritual support for patients | x | x | x | x |  |  |
| 1. Experiencing unprocessed grief | x | x |  | x |  | x |
| 1. Facilitating the last communications between patients and their family | x | x | x | x | x | x |
| 1. Finding positive meaning |  |  | x | x |  |  |
| 1. Large-scale death | x | x |  |  | x | x |
| 1. Managing patients' belongings after death |  | x | x | x | x |  |
| 1. Participating in the information of the patient's death |  | x |  |  |  |  |
| 1. Performing rapid procedures for ascertaining death and preparing the body | x | x | x | x | x | x |
| 1. Providing emotional support to patients and their families | x | x | x | x |  | x |
| 1. Providing end-of-life nursing care to patients | x |  | x | x |  | x |
| 1. Replacing the family in the last farewell | x | x | x | x |  | x |
| 1. Suffering from emotional distress | x | x | x | x | x | x |
| 1. Suffering from moral distress |  | x | x | x | x | x |
| 1. Suffering over disruption of rituals associated with death | x | x |  | x | x | x |
| 1. Unpredictable death | x | x | x | x | x | x |

FG= focus group; x= indicates the subcategory observed.

Note: in grey the focus group in which subcategory is represented for the first time
